# Sevoflurane Inhibits Nuclear Factor-κB Activation in Lipopolysaccharide-Induced Acute Inflammatory Lung Injury via Toll-Like Receptor 4 Signaling

**DOI:** 10.1371/journal.pone.0122752

**Published:** 2015-04-14

**Authors:** Xi Jia Sun, Xiao Qian Li, Xiao Long Wang, Wen Fei Tan, Jun Ke Wang

**Affiliations:** Department of Anesthesiology, First Affiliated Hospital, China Medical University, Shenyang 110001, Liaoning, China; French National Centre for Scientific Research, FRANCE

## Abstract

**Background:**

Infection is a common cause of acute lung injury (ALI). This study was aimed to explore whether Toll-like receptors 4 (TLR_4_) of airway smooth muscle cells (ASMCs) play a role in lipopolysaccharide (LPS)-induced airway hyperresponsiveness and potential mechanisms.

**Methods:**

*In vivo*: A sensitizing dose of LPS (50 µg) was administered i.p. to female mice before anesthesia with either 3% sevoflurane or phenobarbital i.p. After stabilization, the mice were challenged with 5 µg of intratracheal LPS to mimic inflammatory attack. The effects of sevoflurane were assessed by measurement of airway responsiveness to methacholine, histological examination, and IL-1, IL-6, TNF-α levels in bronchoalveolar lavage fluid (BALF). Protein and gene expression of TLR_4_ and NF-κB were also assessed. *In vitro*: After pre-sensitization of ASMCs and ASM segments for 24h, levels of TLR_4_ and NF-κB proteins in cultured ASMCs were measured after continuous LPS exposure for 1, 3, 5, 12 and 24h in presence or absence of sevoflurane. Constrictor and relaxant responsiveness of ASM was measured 24 h afterwards.

**Results:**

The mRNA and protein levels of NF-κB and TLR_4_ in ASM were increased and maintained at high level after LPS challenge throughout 24h observation period, both *in vivo* and *in vitro*. Sevoflurane reduced LPS-induced airway hyperresponsiveness, lung inflammatory cell infiltration and proinflammatory cytokines release in BALF as well as maximal isometric contractile force of ASM segments to acetylcholine, but it increased maximal relaxation response to isoproterenol. Treatment with specific NF-κB inhibitor produced similar protections as sevoflurane, including decreased expressions of TLR_4_ and NF-κB in cultured ASMCs and improved pharmacodynamic responsiveness of ASM to ACh and isoproterenol.

**Conclusions:**

This study demonstrates the crucial role of TLR_4_ activation in ASMCs during ALI in response to LPS. Sevoflurane exerts direct relaxant and anti-inflammatory effects *in vivo* and *in vitro* via inhibition of TLR_4_/NF-κB pathway.

## Introduction

Airway smooth muscle (ASM) is the primary effector tissue for regulating bronchomotor tone. ASM damage occurs in several pulmonary diseases such as asthma and chronic obstructive pulmonary disease (COPD) [1,2]. ASM cells (ASMCs) are highly plastic and their contractility and regulatory functions are altered by cytokines and chemokines released in response to inflammatory stimuli. Recent studies have shown that ASMCs are highly activated during inflammation and lung tissue damage with engagement of Toll-like receptors (TLRs) [3, 4]. TLRs are transmembrane pattern recognition receptors. As a part of the innate immune system, they are key elements in recognizing viral and bacterial components [5, 6]. To date, 11 different TLRs have been identified in humans. All TLRs possess a common intracellular TIR domain for initiating a signal following activation by bacterial components or conserved pathogen-associated molecular patterns of microbes [7, 8]. Detection of microbes by TLRs evokes an inflammatory response. TLR_4_ can be specifically activated by presenting a pathogen-derived antigen to naive T cells when sensing lipopolysaccharide (LPS), a common constituent in the cell wall of gram-negative bacteria, to initiate an immune response [6, 9–12]. Collectively, these data support an emerging concept that the quantity of TLR_4_ expressed on ASMCs will elicit constrictor and relaxant responses of ASM secondary to the autocrine actions of cytokines released by the sensitized ASM itself [2, 13, 14].

It has been well documented that volatile anesthetics contribute to immunosuppression in the postoperative period, especially when they are applied at higher concentrations or for longer times [15]. Volatile anesthetics are used to treat status asthmaticus; thus, the effects of volatile anesthetics on lung tissue have been a focus of attention. Some studies reported that volatile anesthetics delivered by mechanical ventilation alleviated lung inflammation by reducing TNF-α and nitric oxide release in rats that had received intratracheal LPS [16–20]. Sevoflurane is commonly used to sedate patients prior to intubation for mechanical ventilation because of its good controllability [21]. Recently, additional advantages of sevoflurane have been reported, including dilation of bronchioles and reduction of bronchial hyperresponsiveness in patients with asthma, indicating that it has lung protective effects [21–23]. However, the mechanism of these effects remains unclear. Reversible inhibition of voltage-dependent calcium channels and decreased intracellular calcium mobilization may result in a decreased concentration of intracellular calcium in response to volatile anesthetics [21, 24].

There has been some evidence that NF-κB acts as a signal mediator in the attenuation of myocardial and cerebral ischemic reperfusion injuries seen in mice treated with sevoflurane [25–27]. However, whether the mechanisms underlying lung protection are the same as those involved in the heart and brain are not known. Furthermore, the role of TLR_4_ expressed on ASMCs is unknown [28, 29]. The focus of our study was to determine the role of TLR in regulating the responsiveness of ASMCs and the sevoflurane-related modifications of LPS-induced lung inflammation and airway hyperresponsiveness in a series of *in vivo* and *in vitro* experiments. This may lead to new and safe therapeutic methods.

## Materials and Methods

### *In vivo*

#### Ethics statement

Female C57BL/6 mice, weighing 20–30 g, aged 8–12 weeks, were obtained from the Animal Facility at the China Medical University. Mice were bred in micro-isolator cages (24°C) under a 12:12 h light–dark cycle with free access to regular chow and water. Animal care and experimental procedures were approved by the Animal Care and Use Committee of the China Medical University.

#### Animals and experimental protocols

**Fig 1:**
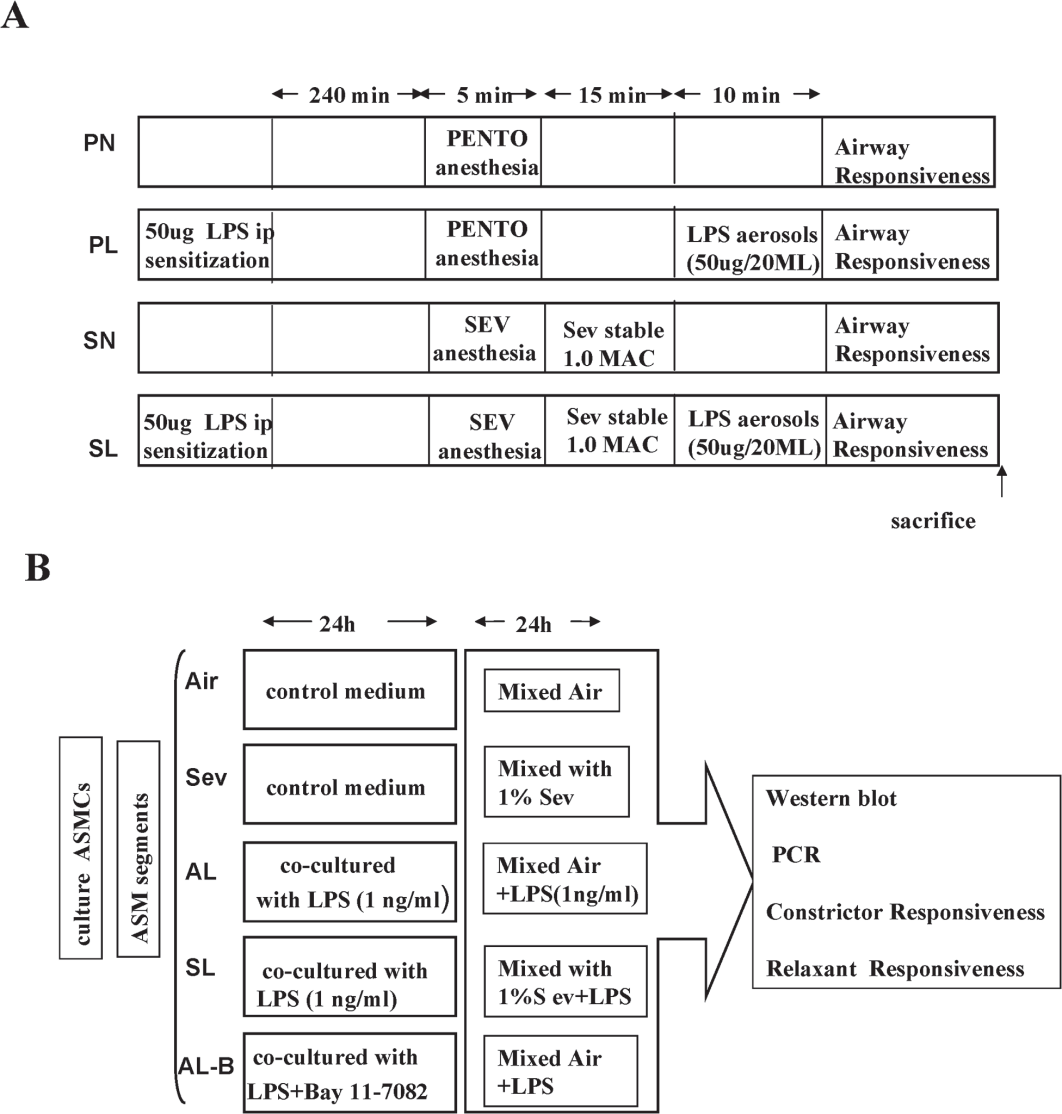
Schematic diagram of experimental design. (**A**) Experimental protocol *in vivo*. (**B**) Experimental protocol *in vitro*.

At 42–56 days of age, all mice were anesthetized with phenobarbital (20 mg/kg, i.p.) 4 hours after treatment with 50 μg LPS (*Escherichia coli* serotype 055:B5; Sigma-Aldrich) for sensitization. After stabilization, the mice were randomized to one of four groups: PENTO + Normal saline (NS) group (PN group, n = 8), PENTO + LPS group (PL group, n = 8), SEV + NS group (SN group, n = 8), and SEV + LPS group (SL group, n = 8), and continuously maintained under anesthesia with 3% sevoflurane (Abbott, Wiesbaden, Germany) or with additional bolus doses of phenobarbital, if necessary. Anesthesia depth was ensured by absent pedal reflexes throughout the experiments. Tracheas were exposed with a neck midline incision under sterile conditions, cannulated with a 20-gauge, 1-inch-long catheter, and sutured. Animals were ventilated at a tidal volume of 20 ml/kg with 100 breaths per minute. The end-tidal sevoflurane concentration was measured continuously with a gas analyzer (Capnomac Ultima; Datex, Helsinki, Finland). After the end-tidal concentration of sevoflurane was stable for 15 min, airway responsiveness to a methacholine (Mch) challenge was evaluated and the mice were euthanized to harvest tissue samples. During surgical and experimental procedures, oxygen saturation, heart rate, and rectal temperature were continuously monitored and maintained within physiologic ranges (Fig 1A).

#### Airway responsiveness determination

Airway responsiveness to methacholine (Mch, acetyl-ß-methylcholine chloride, Sigma, St. Louis) was assessed with a plethysmographic chamber (Buxco Electronics, Sharon, CT) sized for mice and connected to a pressure transducer (PT-5; Grass Instruments, West Warwick, RI). Mice were exposed to increasing concentrations of nebulized Mch (2.5 μg/kg, 25 μg/kg, 50 μg/kg, 100 μg/kg, and 200 μg/kg) by an aerosonic ultrasonic nebulizer (DeVilbiss). The initial values were taken as baseline and the expiratory resistance (Re) and dynamic lung compliance (Cldyn) values for each Mch challenge dose were recorded with AniRes2003 software. The time interval of administration was at least 5 min to ensure the pressure returned to baseline.

#### Harvesting and analysis of BALF

Lung inflammation was evaluated by cell counts and protein contents in bronchoalveolar lavage fluid (BALF). The left lung was ligated and the right lung was lavaged three times with 1 mL of PBS. The amount of BALF retrieved was approximately 80% of the instilled volume. After centrifugation for 10min at 1000r/min, the pellet was resuspended in PBS. Total cell numbers were counted using a hemocytometer, and the numbers of eosinophils, neutrophils, macrophages, and lymphocytes determined on the basis of morphologic criteria. At least 200 cells were counted per sample. The supernatants were collected and immediately frozen on dry ice and stored at -80°C for cytokine measurements using ELISA Kits (Quantikine M; R&D Systems Europe) for IL-1, IL-6, and TNF-α.

#### Lung histopathological examination

After the right hilum was clamped, the left ventricle was punctured at the cardiac apex and slowly lavaged with NS for about 30 min until the fluid outflow from the right auricle was clear. Left lung tissues were then excised and preserved in 10% neutral-buffered formalin, embedded in paraffin, and sectioned into 4-mm-thick slices according to the standard procedure. The sections were deparaffinized, hydrated gradually, and stained with hematoxylin and eosin. The slices were examined by light microscopy.

#### Immunohistochemical analysis for the expression of TLR_4_

The localization of TLR_4_ was determined by immunocytochemistry. Paraffin histologic sections were dewaxed with xylene and then rehydrated in graded concentrations of ethanol. Sections were incubated with rabbit anti-mouse TLR_4_ antibodies (eBioscience, San Diego, CA) overnight at room temperature. Secondary swine anti-rabbit antibodies (DAKO, Copenhagen, Denmark) conjugated to streptavidin-biotin horseradish peroxidase were added for 30 minutes. Staining was revealed using a HRP-Dab Staining Kit (Vector Laboratories, Burlingame, CA). Densitometric analysis was performed using MetaMorph/Evolution MP 5.0/BX51 Image analytical system (Beijing, China).

#### Western blot analysis for TLR_4_ and NF-κB expression

The expression of TLR_4_ and NF-κB was determined by western blot analyses. Cytosolic proteins and nuclear proteins for TLR_4_ or NF-κB were isolated from the lung tissue using a modification of the method described by Dong [30]. The proteins were probed with primary TLR4 antibodies and NF-κB antibodies (Santa Cruz Biotechnology) overnight at 4°C. Then, alkaline phosphatase–conjugated secondary antibodies were added to enhance chemiluminescence detection (NBT/BCIP, Promega, ShangHai, China). Blots were compared with reference to the findings for GAPDH and quantified by densitometric analysis with Lab works software (UVP Upland, CA, USA).

#### Real-time quantitative PCR for TLR_4_ mRNA detection

Total RNA was isolated with TRIzol reagent and purified from ASM by the RNeasy Mini Kit (Qiagen, West Sussex, U.K.) according to the manufacturer’s instructions. Quantitative real-time PCR for TLR_4_ mRNA was carried out using an ABI Prism 7000 (Applied Biosystems, Foster City, CA) under the following amplified cycling conditions: 2 min at 50°C for Uralic-DNA glycosylase incubation; 10-min denaturation at 95°C; 40 cycles of 15-s annealing at 95°C and elongation for 1 min at 60°C with SYBR Green PCR Master Mix Reagent (Applied Biosystems). The primer sequences used for PCR were as follows: TLR_4_: Forward 5'-AGGTCGGTGACTTCAAGAC-3'; Reverse 5'-CCACCTCTGTTTTA-3' NF-κB: Forward 5'-CCTGCTTCTGGAGGGTGATG-3'; Reverse 5'-TCCGGCCGCTATATGCA-3'. To confirm appropriate amplification, the size of the PCR products was verified on gels. The quantity of gene expression was assessed by the comparative Ct method and expression of GAPDH was used as a loading control. All reactions were performed in triplicate.

### *In vitro*

#### ASMC culture and protocols of sevoflurane and LPS exposure

In order to avoid the complexity of *in vivo* models and to better understand the underlying mechanisms, we have simulated the interventions described above in an *in vitro* model. ASMCs were purchased from PriCells (MIC-CELL-0005, PriCells, WuHan, China). They were cultured and expanded in Dulbecco’s modified Eagle’s medium (DMEM) supplemented with 10% fetal bovine serum according to the manufacturer’s instructions. Cells at passages 4–6 were used for experiments.

The ASMCs were plated at a density of 3 × 10^5^ cells in airtight glass chambers, the entrances of which contained calibrated vaporizers (Draeger, Lübeck, Germany) for humidified air or volatile anesthetics. The concentrations of sevoflurane were monitored by a gas chromatograph (GC-17A; Shimadzu, Kyoto, Japan) at the exit port of the chamber. The cells were incubated with serum-free DMEM for 24 h and then treated with different protocols as follows: The ASMCs were incubated for 24 h at room temperature in control or LPS-containing medium, continuously stimulated with LPS (1 ng/ml) to induce cytokine secretion and to mimic an inflammatory response, in the presence or absence of 10 μM Bay 11–7082, a specific NF-κB inhibitor (Alexis Biochemical, San Diego, CA). In parallel experiments, in order to determine the relationship between duration of exposure to LPS and the effects of sevoflurane on cytokine secretion of ASMCs, LPS-stimulated ASMCs were exposed to either air or volatile anesthetics by directing a 95% air-5% CO_2_ mixture through volatile anesthetic vaporizers attached to the entrance of the chamber in the presence or absence of 1% sevoflurane. Samples of culture media and cells for analysis were taken from both treated and control groups after sevoflurane exposure for 1, 3, 5, 12, or 24 h. Each experimental condition was performed in duplicate (Fig 1B).

#### Immunoblot analysis of TLR_4_ and NF-κB

TLR_4_ and NF-κB in lysates isolated from cultured ASMCs were detected using the ECL system (Amersham Biosciences, US) followed by exposure to autoradiography film. The protein band intensities were quantified by densitometry as previously described.

#### Real-time quantitative PCR for TLR_4_ mRNA detection

TLR4 mRNA in purified nuclear samples from cultured ASMCs as assessed by real-time quantitative PCR performed as above.

### Preparation and treatment of mouse ASM tissues

After general anesthesia with intramuscular injections of phenobarbital (20 mg/kg) and exsanguination, tracheas were dissected via open thoracotomy and divided into eight ring segments, 6–8 mm in length, as described previously [31]. The ASM segments were cultured in Krebs-Henseleit buffer at room temperature for 24 h and then treated according to the same protocols as cultured ASMCs above.

#### Pharmacodynamic studies of ASM constrictor and relaxant responsiveness

After 24-h incubation, ASM segments were placed in organ baths containing Krebs-Henseleit buffer aerated with 5% CO_2_ in oxygen, and the tissues were attached to force transducers (Trading, Aarhus, Denmark) from which isometric tension was continuously displayed on a multichannel recorder. Tracheal segments were mounted on two L-shaped metal prongs, with a resting tension of 0.8 mN, as previously described [32]. The maximal isometric contractile force (Tmax) of ASM segments to cumulative administration of acetylcholine (ACh) in final bath and subsequently maximal relaxation (Rmax) to isoproterenol were assessed as previously described [32]. The relaxation responses to isoproterenol were elicited after half-maximal contraction by an ED_50_ dose of ACh and analyzed in terms of % Rmax from the initial contracted level. Concentrations of ACh from10^-9^ to 10^–3^ M and of isoproterenol from 10^–9^ to 10^–4^ M were used in this experiment.

### Statistical analysis

Data are presented as mean ± standard error of mean (SEM). Comparisons between groups were made using the Student’s t-test (two-tailed) or ANOVA with Tukey’s post-test analysis, where appropriate. All test results were considered significant at *P* < 0.05.

## Results

### Airway responsiveness determination

**Fig 2:**
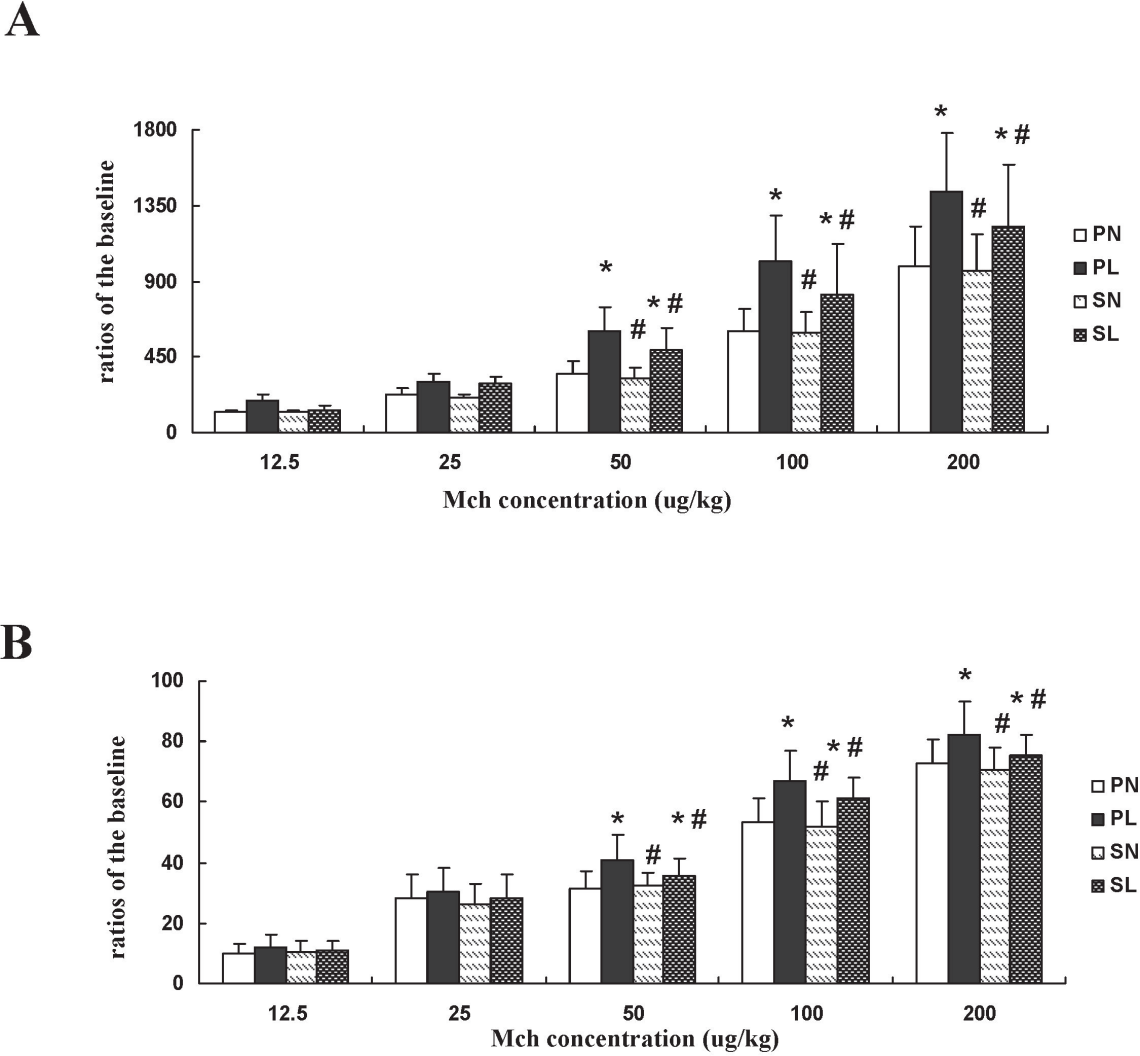
Effects of sevoflurane on expiratory resistance (Re) and dynamic lung compliance (Cldyn) in mice after different concentrations of methacholine (Mch) after LPS challenge. (**A**) Ratios of Re changed with different concentrations of Mch. **(B**) Ratios of Cldyn changed with different concentrations of Mch. Data are presented as means ± SEM (n = 8 per group). *P < 0.05 versus PN; #*P* < 0.05, versus PL.

Airway responsiveness was calculated as the ratio of expiratory resistance (Re) and dynamic lung compliance (Cldyn) following exposure to different Mch doses. Re was significantly increased and Cldyn significantly decreased in mice pretreated with LPS at each challenge dose of Mch, whether in the absence or presence of sevoflurane (Fig 2A, *P* < 0.05). The changes in Re and Cldyn in mice with LPS exposure were significantly higher than the changes in those anesthetized with sevoflurane when challenged with doses of 50 μg/kg, 100 μg/kg, and 200 μg/kg (*P* < 0.05). There were no significant differences among the other groups (Fig 2B, *P* > 0.05).

### Histologic assessment, protein content, and cellular recruitment in BALF

**Fig 3:**
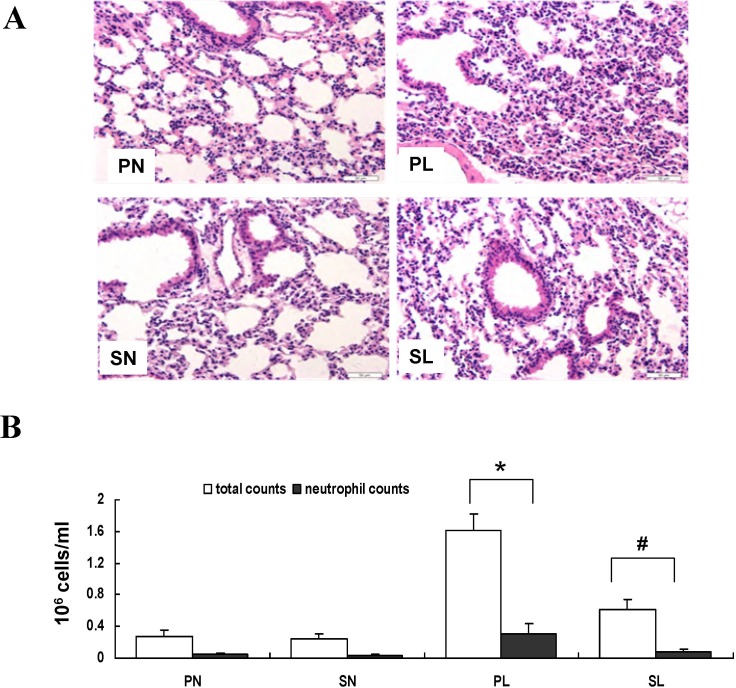
Effects of sevoflurane on histopathologic outcome and cell counts in bronchoalveolar lavage fluid (BALF) after LPS challenge *in vivo*. (**A**) Representative photomicrographs of lung sections stained with hematoxylin-and-eosin from mice anesthetized with phenobarbital or sevoflurane in the absence or presence of LPS challenge. The mice in group PN and SN showed much less inflammatory cell infiltration, alveolar septal thickening, and pulmonary edema in the lung compared with the mice exposed to LPS (NL+SL). Sevoflurane administered after intraperitoneal sensitization could mitigate lung inflammation upon re-challenge with LPS. Original magnification 100×. (**B**) Total cell and neutrophil counts in BALF. Data are presented as means ± SEM (n = 8 per group).*P < 0.05 versus PN; #*P* < 0.05, versus PL.

We found that there were marked increments in neutrophil infiltration, interstitial edema, alveolar septal thickening, and airway smooth muscle damage in mice subjected to LPS sensitization, compared to controls. Continuously inhaled sevoflurane clearly ameliorated these histopathological changes that accompanied lung inflammation (Fig 3A, *P* < 0.05).

Additionally, exposure to LPS increased the protein content and neutrophil recruitment in BALF compared to that observed in controls. Sevoflurane inhaled after LPS sensitization decreased cellular and neutrophil recruitment into BALF and the protein content increase (Fig 3B, *P* < 0.05).

### Cytokine measurement in BALF

Proinflammatory factors such as IL-1, IL-6, and TNF-α have been implicated as important mediators of inflammatory events. LPS challenge increased levels of IL-1, IL-6, and TNF-α compared with the controls, regardless of anesthetic techniques. Treatment with sevoflurane after intraperitoneal sensitization halted the increase of the above factors after LPS aerosol challenge (Table 1, *P* < 0.05).

**Table 1:** Effects of sevoflurane on concentrations of cytokines in BALF of mice exposed to LPS (ng/L).

	PN	PL	SN	SL
IL-1	21.3±2.2	51.1±5.3*	20.5±1.9#	38.4±4.5* #
IL-6	23.3±2.5	102.8±7.1*	24.5±2.2#	89.6±6.5* #
TNF-α	25.4±3.2	85.2±5.8*	26.5±4.1#	63.9±4.7* #

Data are presented as mean ± SD (n = 8 per group).

*P<0.05 compared with PN;

# P<0.05 compared with PL.

### Measurement of TLR_4_ in lung tissue

**Fig 4:**
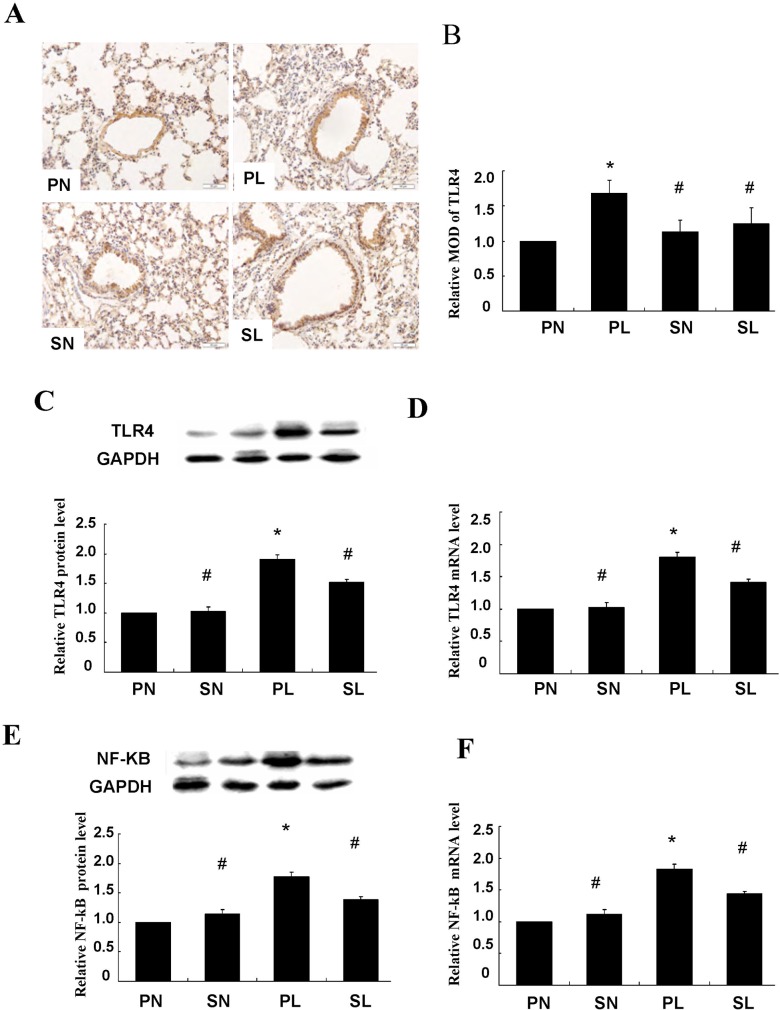
Immunohistochemical staining of TLR_4_ in trachea and effects of sevoflurane on TLR_4_ protein and mRNA expression after LPS challenge *in vivo*. (**A**) Representative micrographs of TLR_4_ in trachea of mice anesthetized with phenobarbital or sevoflurane in absence or presence of LPS challenge. The presence of TLR_4_ was observed only in the smooth muscle cell layer, whose cell membrane and cytoplasm stained tan as detected by horseradish peroxidase–labeled antibodies. Scale bars = 50 μm. (**B**) Histogram for quantification of TLR_4_ in smooth muscle cell layer. The mean optical density values (MOD) were calculated after normalizing against PN. (**C**) Representative western blot and quantitative analysis of TLR_4_ isolated from smooth muscle cell layer after LPS challenge. (**D**) Quantitative real-time PCR of TLR_4_ mRNA expressions in isolated smooth muscle cell layer. (**E**) Representative western blot and quantitative protein analysis of NF-κB in nuclear extracts from smooth muscle cell layer after LPS challenge. (**F**) Quantitative real-time PCR of NF-κB mRNA expressions in smooth muscle cell layer after LPS challenge. Sevoflurane prevented significant increases in protein and mRNA expressions of TLR_4_ and NF-κB in isolated smooth muscle cell layer from mice after LPS challenge. The relative integral density values (IDVs) were calculated after normalizing against GAPDH in each sample and presented as relative protein expression units. Data are presented as means ± SEM (n = 8 per group). *P < 0.05 versus PN; #*P* < 0.05, versus PL.

Immunoreactivity of TLR_4_ was observed only in the cell membrane and cytoplasm of the smooth muscle cell layer (Fig 4A). Quantitative analysis showed that sections taken from the mice exposed to LPS were stained more extensively and deeper than those in control groups. Immunoreactivity of TLR_4_ in response to LPS was decreased after pretreatment with sevoflurane (Fig 4B, *P* < 0.05). Activation of TLR_4_ in the smooth muscle layer was closely related to LPS-induced lung injury, and could be attenuated by sevoflurane inhalation. These findings were further confirmed and quantified by western blotting and real-time PCR (Fig 4C and 4D, *P* < 0.05).

### Measurement of NF-κB in lung tissue

The TLR signaling pathway culminates in the activation of the transcription factor NF-κB. We examined whether up-regulation of TLR_4_ would lead to NF-κB activation and whether treatment with sevoflurane affected such a change. Western blot analysis indicated that markedly higher levels of NF-κB protein were expressed in mice exposed to LPS than in the controls, and this closely accompanied the up-regulation of TLR_4_ (Fig 4D, *P* < 0.05). Additionally, these changes were ameliorated by sevoflurane preconditioning between sensitization and re-challenge (Fig 4D, *P* < 0.05).

Similar results were found with real-time PCR (Fig 4E, *P* < 0.05). These results suggest that TLR_4_ and NF-κB are likely involved in the same signaling pathway, and the inhibition of NF-κB translocation mostly contributed to the protective effects of sevoflurane. However, it was interesting to find that sevoflurane alone, in the absence of LPS challenge, did not affect either TLR_4_ or NF-κB expression.

### Effects of sevoflurane on protein expression of TLR_4_ and NF-κB in isolated ASMCs

**Fig 5:**
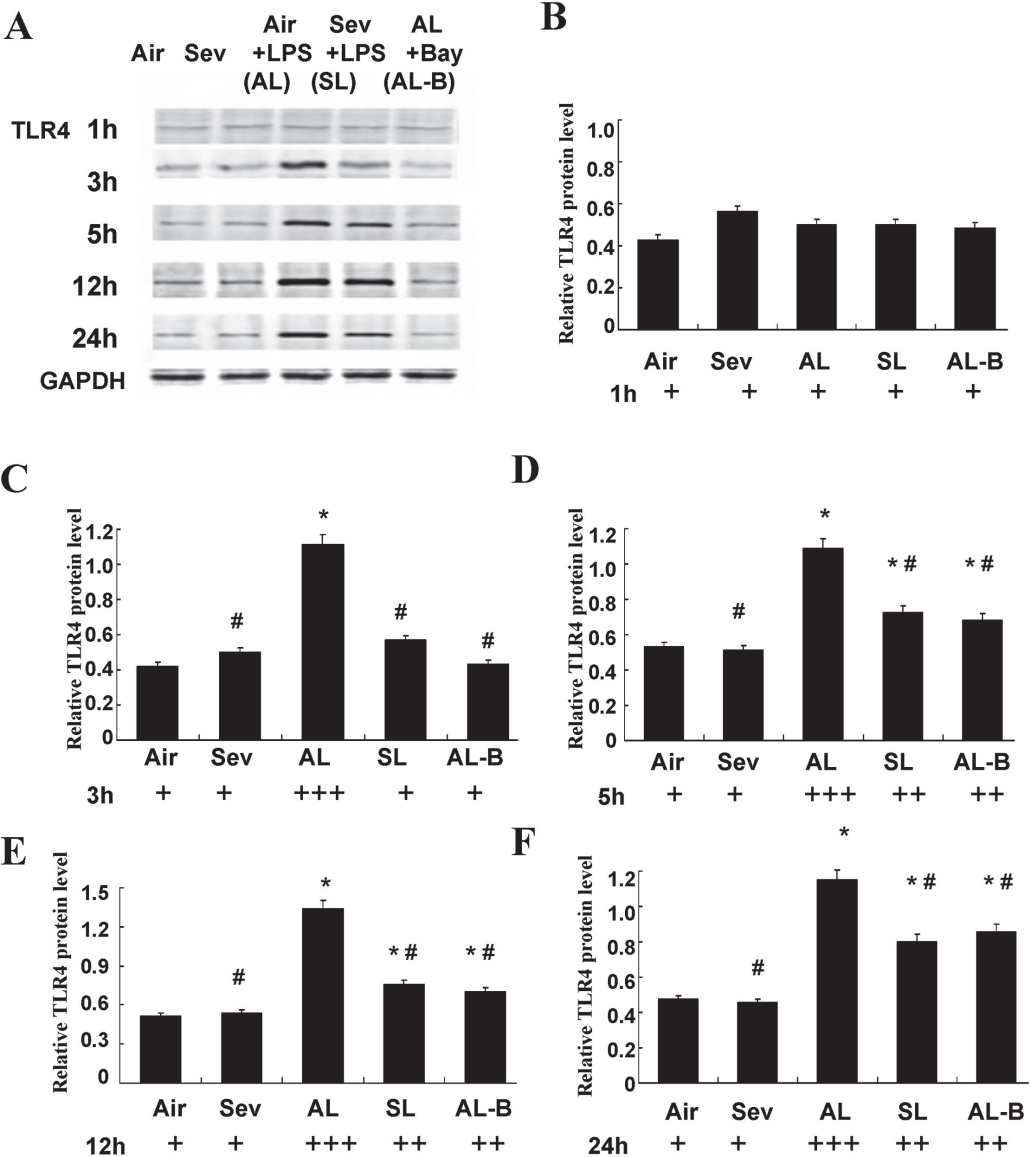
Effects of sevoflurane on TLR_4_ protein expression in airway smooth muscle cells (ASMCs) after continuous LPS exposure for 1, 3, 5, 12, and 24 h *in vitro*. (**A**) Representative western blot of TLR_4_ in cultured ASMCs under different protocols. (**B-F**) Quantitative protein analysis of TLR_4_ in cultured ASMCs under different protocols. The relative integral density values (IDVs) were calculated after normalizing against GAPDH in each sample and presented as relative protein expression units. Sevoflurane prevented TLR_4_ increases in ASMCs at 3, 5, 12, and 24 h after continuous LPS exposure. Data are presented as means ± SEM. * *P* < 0.05 versus Group Air; #*P* < 0.05 versus AL.

**Fig 6:**
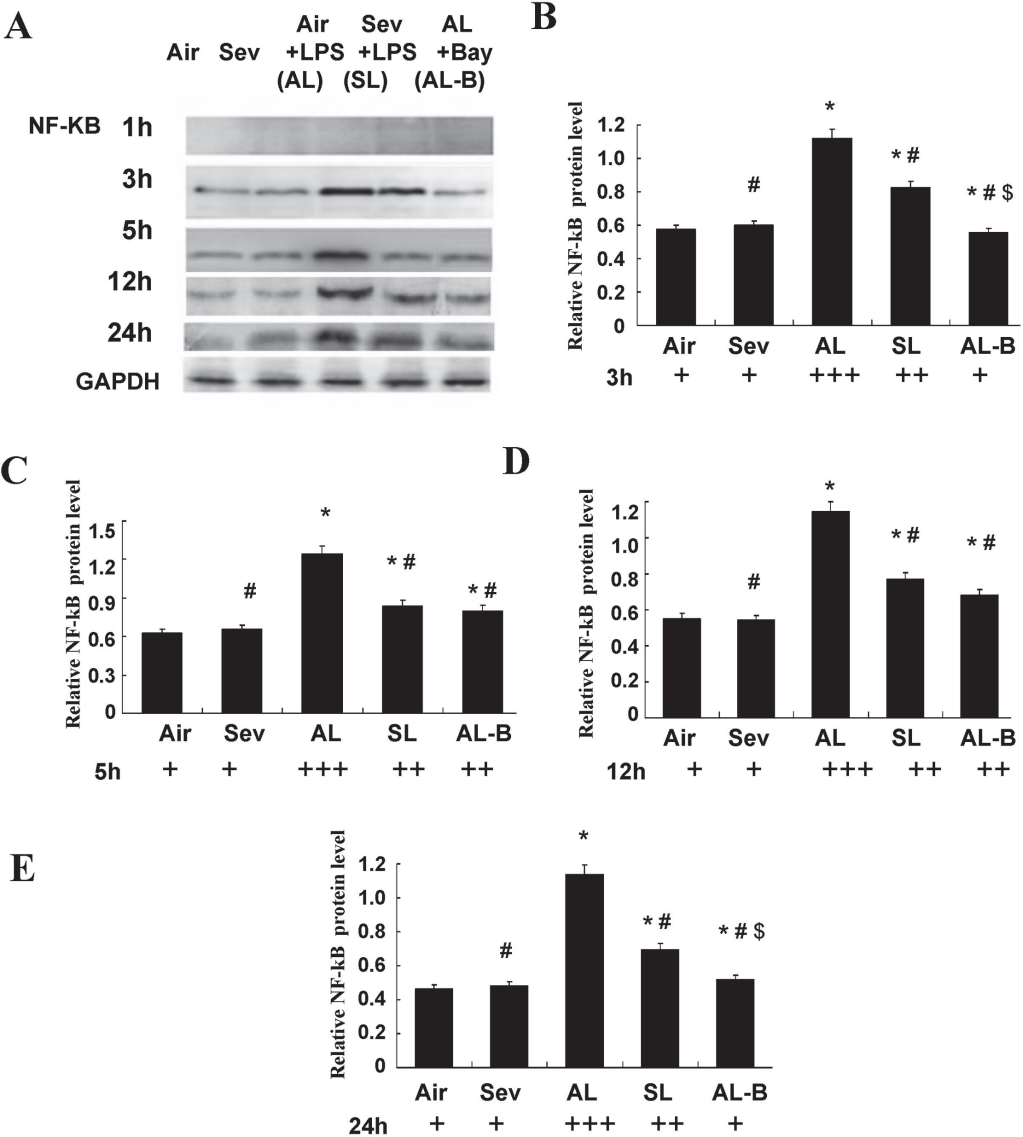
Effects of sevoflurane on NF-κB protein expression in nuclear extracts of airway smooth muscle cells (ASMCs) after continuous LPS exposure for 1, 3, 5, 12, and 24 h *in vitro*. (**A**) Representative western blot of NF-κB in cultured ASMCs under different protocols. (**B-E**) Quantitative protein analysis of NF-κB in cultured ASMCs under different protocols. The relative integral density values (IDVs) were calculated after normalizing against GAPDH in each sample and presented as relative protein expression units. Sevoflurane prevented NF-κB increases in ASMCs at 3, 5, 12, and 24h after continuous LPS exposure. Data are presented as means ± SEM. * *P* < 0.05 versus Group Air; #*P* < 0.05 versus AL; $*P* < 0.05 versus SL.

We performed parallel experiments in cultured ASMCs. As shown in Figs 5 and 6, significantly increased protein expression of TLR_4_ and NF-κB was measured after LPS pre-sensitization of ASMCs and continuous LPS exposure (1 ng/ml) for 3, 5, 12, and 24 h (Group Air vs. Group AL, *P* < 0.05). Sevoflurane prevented these increases in TLR_4_ and NF-κB (Group AL vs. Group SL, *P* < 0.05). There were no marked changes of TLR_4_ and NF-κB in cultured ASMCs at the above time points after treatment with sevoflurane or in controls without LPS challenge (Group Air vs. Group Sev, *P* > 0.05).

We performed experiments with Bay 11–7082 (10 μM), a specific inhibitor of NF-κB, to further confirm the signal mediating activation of TLR_4_. The results showed synergistically protective actions to decrease the expression of TLR_4_ and NF-κB throughout the 24 h observation period (Figs 5 and 6).

### ASM constrictor and relaxant responsiveness *in vitro*

As a final assessment of the hypothesized interactions between NF-κB and sevoflurane, we compared the constrictor and relaxation responses in LPS-exposed isolated ASM segments in the absence and presence of sevoflurane and NF-κB inhibitors. Relative to control ASM, the maximal isometric contractile force responses to ACh were significantly increased in LPS-exposed rings (T_max_ = 112.2 ± 8.4 g/g vs. 85.3 ± 8.6 g/g in control tissues, *P* < 0.05). The enhanced responsiveness to ACh was mitigated in rings pretreated with sevoflurane (Tmax = 91.9 ± 9.9 g/g, *P* < 0.05). Similar protective effects were observed after pretreatment with Bay 11–7082 (96.9 ± 7.3 g/g, *P* > 0.05).

Under the same treatment conditions, the relaxation responses to isoproterenol were significantly attenuated in LPS-exposed ASM, in which the mean Rmax responses amounted to 34.8 ± 4.7% vs. 59.1 ± 3.8% obtained in the control tissues (*P* < 0.05). The decreased ratios of Rmax were significantly mitigated in SL after LPS-challenge to 42.3± 3.6% (*P* < 0.05), and treatment with Bay 11–7082 provided similar Rmax values as the SL group (44.2± 4.0%, *P* > 0.05). Additionally, there were no marked changes in Tmax and Rmax in ASM of controls, whether or not they were treated with sevoflurane (*P* > 0.05) in the absence of LPS-challenge.

## Discussion

It has been noted that surgical patients are more prone to airway hyperreactivity (AHR) during the perioperative period if they have previously had a respiratory infection. A better knowledge of the role of infection in AHR exacerbations might reveal new therapeutic interventions. TLR_4_ is ubiquitous in cells and is specifically activated by LPS. LPS is a major initiator of host immune responses and triggers expression of TLR_4_ [24] and precipitates lung injury [7, 10]. Therefore, intratracheal LPS instillation provides a useful experimental system for investigating the mechanisms of AHR in anesthetized and ventilated rats. Our study is among the first to demonstrate the effectiveness of sevoflurane inhalation in modifying AHR by interfering with the activation of NF-κB via TLR_4_ on ASM after LPS-sensitization.

BALB/c mice were first treated with 50 μg LPS i.p. to initiate local inflammatory reactions. The dosage of LPS and length of exposure were determined from preliminary experiments. There were no significant differences among the groups if the dose was too small or the exposure time was too short, whereas at a high dose or for a long time, ALI occurred during the sensitization period instead of during airway exposure. As previously reported, pulmonary function was obviously altered at 4 h after endotoxin administration in a mouse model of endotoxin-induced lung injury [33]. This is the time period we chose for sensitization. In our model, we observed lung function alteration by airway responsiveness to methacholine after LPS exposure in the presence or absence of inhaled sevoflurane. The results of the present study showed that prolonged stimulation of ASM with LPS resulted in a marked increase in airway responses to methacholine.

TLR_4_ has been demonstrated to be a specific receptor for LPS. To further identify the role of TLR_4_ after LPS exposure, the presence of TLR_4_ was revealed in the smooth muscle cell layers by immunohistochemistry. Histological examination of lung tissues showed significant deterioration in mice with up-regulated TLR_4_ expression compared to controls after LPS exposure, which is consistent with the role of TLR_4_ in the regulation of immunity, as has been found in previous studies. Some studies showed a positive correlation with the level of TLR_4_ expression and the extent of LPS-induced inflammatory cell recruitment in the airways [16, 30]. The severity of AHR and pulmonary fibrosis was decreased and the prognosis improved by inhibiting TLR_4_ expression [6, 8, 29, 34]. All of these data indicate that the activation of TLR_4_ on ASMs is important in the pathogenesis of lung inflammation after LPS exposure. The severity of lung inflammation is closely related to AHR (Fig 3 and Table 1).

Sevoflurane is one of the most commonly used volatile anesthetics. Additionally, sevoflurane produces anti-inflammatory and bronchiodilatory effects by unknown mechanisms [21–23]. Some studies showed that sevoflurane inhibited neutrophil function and the production of reactive oxygen species (ROS) in ischemic reperfusion injuries [25, 35]. Sevoflurane was also found to suppress pro-inflammatory cytokine production and inducible NO synthase/NO biosynthesis in LPS-activated macrophages [17, 36]. With this in mind, we conducted this study of sevoflurane aimed to investigate the effect of sevoflurane on the expression of TLR_4_ in lung tissue, since elevated expression levels of TLR_4_ were closely associated with the extent of the acute pulmonary response to inhaled endotoxin. Our investigation strongly supported the anti-inflammatory effects of sevoflurane in ALI: sevoflurane improved LPS-induced ALI *in vivo* by improving lung histological alteration, decreasing inflammatory cytokine levels in BALF, and inhibiting TLR_4_ gene and protein expressions in lung tissue. Similar changes of NF-κB were detected by western blotting and real-time PCR. Volatile anesthetics have been reported to reduce the LPS-induced inflammatory responses in airway smooth muscle by opposing the actions of ERK_1/2_ and p38 MAPK signaling [3, 34]. Thus, we hypothesized that TLR_4_ was essential in the development of airway inflammation and that the critical role of TLR_4_ was most likely to be mediated via NF-κB signaling pathway and reversed by sevoflurane inhalation.

Epithelial cells and smooth muscle cells are the first to encounter invading microbes. There is increasing evidence demonstrating that ASMCs are not simple contractile elements, but also modulate immunity by releasing different cytokines and chemokines. Such a response could also be mediated via the release of inflammatory mediators from the bronchial epithelium and/or inflammatory airway cells known to express a variety of TLR_4_, such as macrophages and neutrophils [30, 34, 37]. In our model, sevoflurane exerted anti-inflammatory effects in lung tissue and bronchial relaxation in isolated bronchial smooth muscle by attenuation of TLR_4_ activation, as illustrated by the changes of cell counts, IL-1, IL-6, and TNF-α levels in BALF and tissues (Fig 3). The above findings were further confirmed and quantified by western blotting and quantitative real-time PCR of TLR_4_ (Fig 4).

Based on this assumption and the results *in vivo*, the second part of our study was designed with protocols *in vitro*. Cultured human airway smooth muscle cells were found to express several TLRs, with a pronounced expression of TLR_2_ and TLR_4,_ as had been demonstrated in mouse lung by real-time PCR analysis previously [10]. These findings validated the *in vitro* use of ASMCs of mouse trachea to further confirm the hypothesis proposed above. The ASMCs were first co-incubated with a small dose of LPS designed to imitate the circumstances of potential inflammatory stimuli. These observations were consistent with our previous *in vivo* study, and showed that both gene and protein expressions of TLR4 and NF-κB in cultured ASMCs were significantly increased at 3, 5, 12 and 24 h after LPS exposure. The protective action of sevoflurane was indicated by its marked inhibition of TLR_4_ and NF-κB mRNA expression and protein expression *in vitro*. Furthermore, we performed additional experiments with a specific inhibitor of NF-κB (Bay 11–7082) to determine whether sevoflurane specifically inhibited NF-κB activation. The results showed that administration of the NF-κB inhibitor presented similar protective effects as sevoflurane with respect to the decreases in TLR_4_ and NF-κB expressions at 3, 5, 12, and 24 h (Figs 5 and 6) and improved the constrictor and relaxant responsiveness of ASM to ACh and isoproterenol at 24 h after exposure.

Studies have found that the mechanisms regulating TLR_4_ expression in response to LPS varies in different tissues and cell types, partly contributing to different microenvironments or extracellular matrices [30, 34, 37]. Therefore, cultured tracheal segments were used in the last part of our study, with the advantage not only in reducing phenotypic alternations of cultured cells and the potentially complex interactions *in vivo*, but also of getting closer to the internal environment that is in contact with indigenous structures of airways. Results from this work showed that neither LPS exposure nor activation of TLR_4_ alone induced a direct contraction of the ASM. Instead, the activation of TLR_4_ induced by LPS went on to play a role in the regulation of alternative splicing of nuclear NF-κB in lung after injury. The protective effects of sevoflurane were shown with specific NF-κB inhibitor, too.

The results showing protective effects of sevoflurane on ALI are of clinical relevance. First, the protection by volatile anesthetics (such as isoflurane) on lung tissue could be observed after exposure to LPS [7, 10], and the expression of TLR_4_ was found in cultured human airway smooth cells [4, 12]. Second, the experimental protocols *in vivo* and *in vitro* were closely related to clinical practice. For example, the mice sensitized with LPS intraperitoneally ahead of sevoflurane treatment simulate patients with potential airway inflammation before undergoing general anesthesia. Third, the concentrations of sevoflurane used in the *in vivo* experiments were comparable to those in the plasma of people during general anesthesia in clinical practice. In addition, in the *in vitro* experiments, the mean concentrations of sevoflurane in the solution (1.0%, 2.0%, and 3.0% in the gas phase) were 0.17, 0.33, and 0.56 mM, respectively [38]. Each concentration of the anesthetic had a close linear correlation with each concentration of the anesthetic in the gas phase. The concentrations of sevoflurane obtained in the culture medium of ASMCs and tracheal segments were 0.41 mM and 0.51 mM, with an equal amount in the gas phase.

## Conclusion

The present study strongly demonstrate the importance of the TLR_4_/NF-κB-dependent signaling cascade for the pathogenesis of airway hyperresponsiveness and inflammatory injury. Sevoflurane exerts direct relaxant and anti-inflammatory effects by decreased histological alterations, decreased inflammatory cytokine release, and decreased responsiveness to Mch and ACh in *vivo* and *in vitro* by inhibiting TLR_4_/NF-κB pathway.
